# *cis*-Cyclo­heptane-1,2-diol

**DOI:** 10.1107/S160053680905051X

**Published:** 2009-11-28

**Authors:** Richard Betz, Peter Klüfers, Peter Mayer

**Affiliations:** a Ludwig-Maximilians Universität, Department Chemie und Biochemie, Butenandtstrasse 5–13 (Haus D), 81377 München, Germany

## Abstract

The title compound, C_7_H_14_O_2_, is a vicinal diol derived from cyclo­heptane with *cis*-orientated hydr­oxy groups. The mol­ecules shows no non-crystallographic symmetry. The O—C—C—O torsion angles of both mol­ecules present in the asymmetric unit [−66.4 (2) and −66.9 (2)°] are similar to those in *trans*-configured cyclo­hexane derivatives (including pyran­oses) as well as *rac*-*trans*-cyclo­heptane-1,2-diol, but smaller than those in *trans*-configured cyclo­pentane derivatives (including furan­oses). In the crystal structure, O—H⋯O hydrogen bonds furnish the formation of sheets parallel to [110].

## Related literature

For the synthesis, see: Becker *et al.* (2001 ▶). For torsion angles of *cis*- and *trans*-configured cyclo­hexane-1,2-diols, see: Sillanpää *et al.* (1984 ▶). For the structure of the corresponding *rac*-*trans*-cyclo­heptane-1,2-diol, see: Betz & Klüfers (2007 ▶). For graph-set analysis, see Bernstein *et al.* (1995 ▶); Etter *et al.* (1990 ▶).
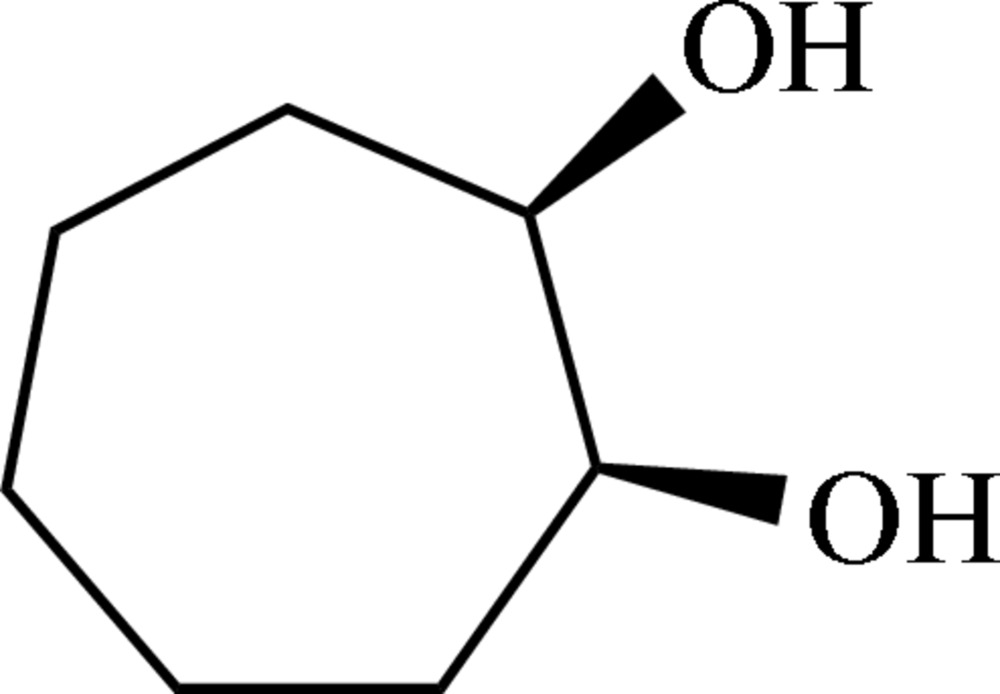


## Experimental

### 

#### Crystal data

C_7_H_14_O_2_
*M* *_r_* = 130.18
Triclinic, 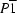

*a* = 7.4148 (5) Å
*b* = 8.7629 (5) Å
*c* = 12.4531 (6) Å
α = 103.861 (4)°
β = 105.685 (4)°
γ = 90.189 (4)°
*V* = 754.32 (8) Å^3^
*Z* = 4
Mo *K*α radiation
μ = 0.08 mm^−1^
*T* = 200 K
0.25 × 0.22 × 0.05 mm

#### Data collection

Nonius KappaCCD diffractometer
4882 measured reflections
2658 independent reflections
1965 reflections with *I* > 2σ(*I*)
*R*_int_ = 0.057

#### Refinement

*R*[*F* ^2^ > 2σ(*F* ^2^)] = 0.069
*wR*(*F* ^2^) = 0.207
*S* = 1.04
2658 reflections
178 parameters
H-atom parameters constrained
Δρ_max_ = 0.65 e Å^−3^
Δρ_min_ = −0.32 e Å^−3^

### 

Data collection: *COLLECT* (Nonius, 2004 ▶); cell refinement: *SCALEPACK* (Otwinowski & Minor, 1997 ▶); data reduction: *DENZO* (Otwinowski & Minor, 1997 ▶) and *SCALEPACK*; program(s) used to solve structure: *SHELXS97* (Sheldrick, 2008 ▶); program(s) used to refine structure: *SHELXL97* (Sheldrick, 2008 ▶); molecular graphics: *ORTEP-3* (Farrugia, 1997 ▶); software used to prepare material for publication: *SHELXL97*.

## Supplementary Material

Crystal structure: contains datablocks I, global. DOI: 10.1107/S160053680905051X/dn2506sup1.cif

Structure factors: contains datablocks I. DOI: 10.1107/S160053680905051X/dn2506Isup2.hkl

Additional supplementary materials:  crystallographic information; 3D view; checkCIF report

## Figures and Tables

**Table 1:** Hydrogen-bond geometry (Å, °)

*D*—H⋯*A*	*D*—H	H⋯*A*	*D*⋯*A*	*D*—H⋯*A*
O11—H11⋯O12^i^	0.84	1.92	2.697 (2)	154
O12—H12⋯O22	0.84	1.90	2.733 (2)	173
O21—H21⋯O11^ii^	0.84	1.88	2.720 (2)	178
O22—H22⋯O21^iii^	0.84	1.91	2.699 (2)	156

Symmetry codes: (i) 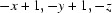; (ii) 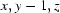; (iii) 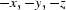.

**Fig. 1.**
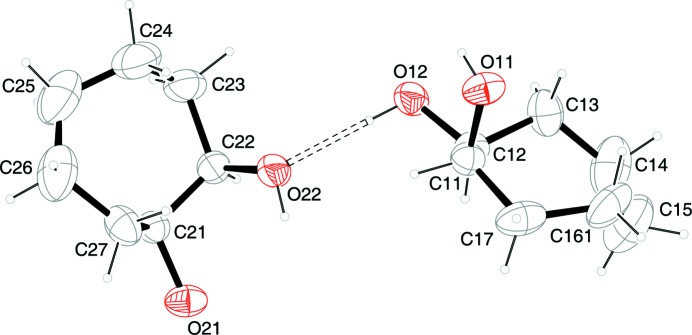
The two molecules comprising the asymmetric unit of (I), with atom labels and anisotropic displacement ellipsoids (drawn at 50% probability level) for non-H atoms. Only the major component of the split C atom (C 16) is shown for clarity.

**Fig. 2.**
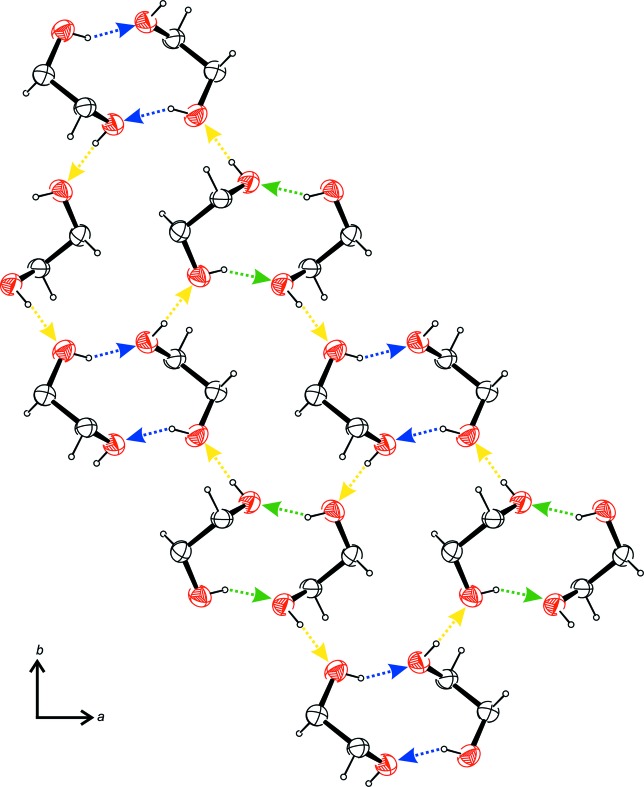
Intermolecular interactions in the crystal structure of the title compound, viewed along [0 0 -1]. For clarity, the carbocyclic moieties were not depicted and the labelling of atoms was replaced by the following colour code: blue arrows indicate hydrogen bonds between the first molecule of the asymmetric unit and its symmetry-generated equivalents, green arrows between the second molecule and its equivalents. Yellow arrows denote hydrogen bonds between the two molecules.

## supplementary crystallographic information

### Comment

During our investigation of the chelation abilities of selected *cis*- and *trans*-configured cyclic vicinal diols and the influence of bonding to various metals, semi-metals and non-metals on the geometry of the chelating molecule, the structure of *cis*-cycloheptane-1,2-diol was determined.

Both molecules present in the asymmetric unit adopt chair-like conformations (Fig. 1). The O–C–C–O torsion angle is found at 60° and 66°, respectively. Both molecules possess (*RS*/*SR*)-configuration.

A disorder of a methylene group in one of the molecules was accounted for by a split model. The major position dominates by a 4:1 ratio.

In the crystal structure, hydrogen bonds furnish the formation of sheets parallel to [1 1 0]. The hydrophobic cycloheptane moieties form the surfaces of these sheets. The description of the hydrogen bonding pattern can be done in two ways: first, it can be seen as a combination of annealated ten- and eighteen-membered rings with diverging directions of rotation. As an alternative, the pattern may be seen as a set of two cooperative, antidromic chains (Fig. 2). In terms of graph-set analysis (Etter *et al.*, 1990; Bernstein *et al.*, 1995), the descriptor on the unitary level is *DDR*^2^_2_(10)*R*^2^_2_(10). While the eighteen-membered rings appear on the ternary level of graph-set analysis with a *R*^6^_6_(18) descriptor, the cooperative chains appear on the quarternary level with a *C*^4^_4_(8) descriptor.

### Experimental

The title compound was prepared by standard procedures upon neutral aqueous dihydroxylation of cycloheptene with potassium permanganate (Becker *et al.*, 2001). Crystals suitable for X-ray diffraction were directly obtained from the solidified reaction product.

### Refinement

All carbon bonded H-atoms were placed in calculated positions (C—H 1.00 Å for methine groups, C—H 0.99 Å for methylene groups) and were included in the refinement in the riding model approximation, with *U*(H) set to 1.2*U*_eq_(C) for both groups. Hydroxyl H atoms were allowed to rotate with a fixed angle around the C—O bond to best fit the experimental electron density (HFIX 147 in the *SHELX* program suite (Sheldrick, 2008)). For the refinement their *U*(H) was set to 1.5*U*_eq_(O).

### Crystal data

**Table:**

C_7_H_14_O_2_	*Z* = 4
*M**_r_* = 130.18	*F*(000) = 288
Triclinic, *P*1	*D*_x_ = 1.146 Mg m^−^^3^
Hall symbol: -P 1	Mo *K*α radiation, λ = 0.71073 Å
*a* = 7.4148 (5) Å	Cell parameters from 8679 reflections
*b* = 8.7629 (5) Å	θ = 3.1–25.0°
*c* = 12.4531 (6) Å	µ = 0.08 mm^−^^1^
α = 103.861 (4)°	*T* = 200 K
β = 105.685 (4)°	Platelet, colourless
γ = 90.189 (4)°	0.25 × 0.22 × 0.05 mm
*V* = 754.32 (8) Å^3^	

### Data collection

**Table:**

Nonius KappaCCD diffractometer	1965 reflections with *I* > 2σ(*I*)
Radiation source: rotating anode	*R*_int_ = 0.057
MONTEL, graded multilayered X-ray optics	θ_max_ = 25.1°, θ_min_ = 3.3°
ω scans	*h* = −8→8
4882 measured reflections	*k* = −10→10
2658 independent reflections	*l* = −14→14

### Refinement

**Table:**

Refinement on *F*^2^	Primary atom site location: structure-invariant direct methods
Least-squares matrix: full	Secondary atom site location: difference Fourier map
*R*[*F*^2^ > 2σ(*F*^2^)] = 0.069	Hydrogen site location: difference Fourier map
*wR*(*F*^2^) = 0.207	H-atom parameters constrained
*S* = 1.04	*w* = 1/[σ^2^(*F*_o_^2^) + (0.1106*P*)^2^ + 0.3333*P*] where *P* = (*F*_o_^2^ + 2*F*_c_^2^)/3
2658 reflections	(Δ/σ)_max_ < 0.001
178 parameters	Δρ_max_ = 0.65 e Å^−^^3^
0 restraints	Δρ_min_ = −0.32 e Å^−^^3^

### Fractional atomic coordinates and isotropic or equivalent isotropic displacement parameters (Å^2^)

**Table:**

	*x*	*y*	*z*	*U*_iso_*/*U*_eq_	Occ. (<1)
O11	0.2847 (2)	0.6535 (2)	0.06402 (15)	0.0429 (5)	
H11	0.3578	0.6190	0.0241	0.064*	
O12	0.4661 (2)	0.36087 (19)	0.06017 (15)	0.0413 (5)	
H12	0.3830	0.2895	0.0191	0.062*	
O21	0.0359 (2)	−0.1787 (2)	−0.05660 (15)	0.0419 (5)	
H21	0.1149	−0.2280	−0.0184	0.063*	
O22	0.2163 (2)	0.11011 (19)	−0.06570 (15)	0.0425 (5)	
H22	0.1434	0.1044	−0.0253	0.064*	
C11	0.2358 (3)	0.5332 (3)	0.1133 (2)	0.0367 (6)	
H111	0.1420	0.4553	0.0512	0.044*	
C12	0.4071 (3)	0.4461 (3)	0.1566 (2)	0.0361 (6)	
H121	0.3713	0.3695	0.1966	0.043*	
C13	0.5755 (4)	0.5519 (3)	0.2380 (2)	0.0507 (7)	
H131	0.6906	0.4971	0.2328	0.061*	
H132	0.5822	0.6488	0.2111	0.061*	
C14	0.5765 (6)	0.5994 (5)	0.3625 (3)	0.0884 (13)	
H141	0.6132	0.5080	0.3953	0.106*	
H142	0.6776	0.6843	0.4024	0.106*	
C15	0.4085 (7)	0.6530 (7)	0.3932 (3)	0.1032 (15)	
H151	0.4474	0.7281	0.4701	0.124*	
H152	0.3392	0.5611	0.4007	0.124*	
C161	0.2784 (8)	0.7289 (5)	0.3149 (3)	0.0689 (17)	0.817 (10)
H161	0.3523	0.7992	0.2885	0.083*	0.817 (10)
H162	0.2013	0.7957	0.3583	0.083*	0.817 (10)
C162	0.192 (2)	0.626 (2)	0.3178 (12)	0.053 (6)	0.183 (10)
H163	0.1397	0.5304	0.3310	0.064*	0.183 (10)
H164	0.1271	0.7155	0.3517	0.064*	0.183 (10)
C17	0.1397 (4)	0.6103 (4)	0.2034 (3)	0.0571 (8)	
H171	0.0835	0.5270	0.2284	0.069*	
H172	0.0362	0.6693	0.1680	0.069*	
C21	0.0931 (3)	−0.1616 (3)	−0.1545 (2)	0.0347 (6)	
H211	0.1280	−0.2659	−0.1940	0.042*	
C22	0.2640 (3)	−0.0448 (3)	−0.1131 (2)	0.0355 (6)	
H221	0.3573	−0.0783	−0.0500	0.043*	
C23	0.3614 (4)	−0.0332 (3)	−0.2039 (3)	0.0536 (8)	
H231	0.4245	−0.1314	−0.2226	0.064*	
H232	0.4600	0.0548	−0.1706	0.064*	
C24	0.2292 (6)	−0.0064 (4)	−0.3191 (3)	0.0742 (10)	
H241	0.1564	0.0856	−0.2997	0.089*	
H242	0.3084	0.0201	−0.3655	0.089*	
C25	0.0944 (6)	−0.1437 (5)	−0.3915 (3)	0.0847 (12)	
H251	0.0633	−0.1368	−0.4726	0.102*	
H252	0.1594	−0.2415	−0.3871	0.102*	
C26	−0.0835 (6)	−0.1586 (5)	−0.3613 (3)	0.0785 (11)	
H261	−0.1345	−0.2698	−0.3925	0.094*	
H262	−0.1743	−0.0940	−0.4015	0.094*	
C27	−0.0755 (4)	−0.1119 (3)	−0.2359 (2)	0.0484 (7)	
H271	−0.1909	−0.1574	−0.2266	0.058*	
H272	−0.0766	0.0043	−0.2122	0.058*	

### Atomic displacement parameters (Å^2^)

**Table:**

	*U*^11^	*U*^22^	*U*^33^	*U*^12^	*U*^13^	*U*^23^
O11	0.0463 (11)	0.0451 (10)	0.0495 (11)	0.0157 (8)	0.0234 (8)	0.0231 (8)
O12	0.0415 (10)	0.0325 (9)	0.0512 (11)	0.0000 (7)	0.0211 (8)	0.0037 (7)
O21	0.0418 (10)	0.0427 (10)	0.0526 (11)	0.0119 (7)	0.0224 (8)	0.0228 (8)
O22	0.0459 (10)	0.0340 (9)	0.0489 (11)	−0.0024 (7)	0.0224 (8)	0.0028 (7)
C11	0.0374 (13)	0.0354 (12)	0.0390 (13)	0.0030 (10)	0.0118 (10)	0.0110 (10)
C12	0.0395 (13)	0.0320 (12)	0.0403 (13)	0.0060 (9)	0.0135 (10)	0.0129 (10)
C13	0.0404 (15)	0.0483 (16)	0.0512 (16)	0.0065 (11)	−0.0014 (12)	0.0054 (12)
C14	0.103 (3)	0.094 (3)	0.0451 (19)	0.008 (2)	−0.0056 (19)	0.0039 (18)
C15	0.117 (4)	0.135 (4)	0.051 (2)	0.013 (3)	0.024 (2)	0.009 (2)
C161	0.109 (4)	0.058 (3)	0.055 (2)	0.030 (3)	0.050 (2)	0.0120 (19)
C162	0.052 (10)	0.074 (13)	0.034 (8)	−0.005 (8)	0.026 (7)	0.000 (7)
C17	0.0635 (19)	0.0571 (17)	0.074 (2)	0.0252 (14)	0.0443 (16)	0.0309 (15)
C21	0.0397 (13)	0.0279 (11)	0.0386 (13)	0.0029 (9)	0.0140 (10)	0.0088 (9)
C22	0.0343 (12)	0.0358 (13)	0.0370 (13)	0.0042 (9)	0.0119 (10)	0.0081 (10)
C23	0.0609 (18)	0.0463 (15)	0.0661 (18)	0.0053 (13)	0.0406 (15)	0.0120 (13)
C24	0.105 (3)	0.069 (2)	0.067 (2)	0.002 (2)	0.049 (2)	0.0249 (17)
C25	0.115 (3)	0.092 (3)	0.0427 (18)	0.014 (2)	0.019 (2)	0.0130 (18)
C26	0.096 (3)	0.079 (2)	0.0438 (18)	0.004 (2)	−0.0042 (18)	0.0103 (16)
C27	0.0437 (15)	0.0467 (15)	0.0489 (16)	−0.0047 (11)	0.0006 (12)	0.0152 (12)

### Geometric parameters (Å, °)

**Table:**

O11—C11	1.431 (3)	C161—H162	0.9900
O11—H11	0.8400	C162—C17	1.343 (15)
O12—C12	1.431 (3)	C162—H163	0.9900
O12—H12	0.8400	C162—H164	0.9900
O21—C21	1.434 (3)	C17—H171	0.9900
O21—H21	0.8400	C17—H172	0.9900
O22—C22	1.431 (3)	C21—C22	1.518 (3)
O22—H22	0.8400	C21—C27	1.520 (3)
C11—C17	1.515 (3)	C21—H211	1.0000
C11—C12	1.523 (3)	C22—C23	1.519 (3)
C11—H111	1.0000	C22—H221	1.0000
C12—C13	1.518 (3)	C23—C24	1.573 (5)
C12—H121	1.0000	C23—H231	0.9900
C13—C14	1.503 (5)	C23—H232	0.9900
C13—H131	0.9900	C24—C25	1.498 (5)
C13—H132	0.9900	C24—H241	0.9900
C14—C15	1.447 (6)	C24—H242	0.9900
C14—H141	0.9900	C25—C26	1.480 (6)
C14—H142	0.9900	C25—H251	0.9900
C15—C161	1.459 (6)	C25—H252	0.9900
C15—C162	1.609 (17)	C26—C27	1.501 (4)
C15—H151	0.9900	C26—H261	0.9900
C15—H152	0.9900	C26—H262	0.9900
C161—C17	1.611 (6)	C27—H271	0.9900
C161—H161	0.9900	C27—H272	0.9900
			
?···?	?		
			
C11—O11—H11	109.5	C162—C17—C161	43.2 (8)
C12—O12—H12	109.5	C11—C17—C161	113.6 (3)
C21—O21—H21	109.5	C162—C17—H171	65.7
C22—O22—H22	109.5	C11—C17—H171	108.8
O11—C11—C17	107.3 (2)	C161—C17—H171	108.8
O11—C11—C12	110.63 (19)	C162—C17—H172	120.6
C17—C11—C12	115.3 (2)	C11—C17—H172	108.8
O11—C11—H111	107.8	C161—C17—H172	108.8
C17—C11—H111	107.8	H171—C17—H172	107.7
C12—C11—H111	107.8	O21—C21—C22	108.75 (19)
O12—C12—C13	106.8 (2)	O21—C21—C27	107.1 (2)
O12—C12—C11	108.94 (19)	C22—C21—C27	114.1 (2)
C13—C12—C11	114.6 (2)	O21—C21—H211	108.9
O12—C12—H121	108.8	C22—C21—H211	108.9
C13—C12—H121	108.8	C27—C21—H211	108.9
C11—C12—H121	108.8	O22—C22—C21	110.90 (18)
C14—C13—C12	115.8 (3)	O22—C22—C23	107.6 (2)
C14—C13—H131	108.3	C21—C22—C23	115.3 (2)
C12—C13—H131	108.3	O22—C22—H221	107.6
C14—C13—H132	108.3	C21—C22—H221	107.6
C12—C13—H132	108.3	C23—C22—H221	107.6
H131—C13—H132	107.4	C22—C23—C24	115.2 (2)
C15—C14—C13	120.0 (3)	C22—C23—H231	108.5
C15—C14—H141	107.3	C24—C23—H231	108.5
C13—C14—H141	107.3	C22—C23—H232	108.5
C15—C14—H142	107.3	C24—C23—H232	108.5
C13—C14—H142	107.3	H231—C23—H232	107.5
H141—C14—H142	106.9	C25—C24—C23	115.1 (3)
C14—C15—C161	117.2 (4)	C25—C24—H241	108.5
C14—C15—C162	130.3 (6)	C23—C24—H241	108.5
C161—C15—C162	42.3 (7)	C25—C24—H242	108.5
C14—C15—H151	108.0	C23—C24—H242	108.5
C161—C15—H151	108.0	H241—C24—H242	107.5
C162—C15—H151	121.1	C26—C25—C24	116.5 (3)
C14—C15—H152	108.0	C26—C25—H251	108.2
C161—C15—H152	108.0	C24—C25—H251	108.2
C162—C15—H152	65.7	C26—C25—H252	108.2
H151—C15—H152	107.2	C24—C25—H252	108.2
C15—C161—C17	115.2 (4)	H251—C25—H252	107.3
C15—C161—H161	108.5	C25—C26—C27	117.4 (3)
C17—C161—H161	108.5	C25—C26—H261	108.0
C15—C161—H162	108.5	C27—C26—H261	108.0
C17—C161—H162	108.5	C25—C26—H262	108.0
H161—C161—H162	107.5	C27—C26—H262	108.0
C17—C162—C15	122.7 (11)	H261—C26—H262	107.2
C17—C162—H163	106.7	C26—C27—C21	115.9 (3)
C15—C162—H163	106.7	C26—C27—H271	108.3
C17—C162—H164	106.7	C21—C27—H271	108.3
C15—C162—H164	106.7	C26—C27—H272	108.3
H163—C162—H164	106.6	C21—C27—H272	108.3
C162—C17—C11	129.7 (8)	H271—C27—H272	107.4
			
O11—C11—C12—O12	−66.4 (2)	O11—C11—C17—C161	−69.0 (3)
C17—C11—C12—O12	171.6 (2)	C12—C11—C17—C161	54.7 (3)
O11—C11—C12—C13	53.2 (3)	C15—C161—C17—C162	49.7 (11)
C17—C11—C12—C13	−68.8 (3)	C15—C161—C17—C11	−73.9 (4)
O12—C12—C13—C14	−155.2 (3)	O21—C21—C22—O22	−66.9 (2)
C11—C12—C13—C14	84.1 (3)	C27—C21—C22—O22	52.5 (3)
C12—C13—C14—C15	−45.7 (5)	O21—C21—C22—C23	170.5 (2)
C13—C14—C15—C161	−28.3 (7)	C27—C21—C22—C23	−70.1 (3)
C13—C14—C15—C162	20.8 (13)	O22—C22—C23—C24	−73.0 (3)
C14—C15—C161—C17	80.7 (6)	C21—C22—C23—C24	51.3 (3)
C162—C15—C161—C17	−40.4 (8)	C22—C23—C24—C25	−69.2 (4)
C14—C15—C162—C17	−31 (2)	C23—C24—C25—C26	84.1 (4)
C161—C15—C162—C17	56.6 (14)	C24—C25—C26—C27	−36.0 (5)
C15—C162—C17—C11	35 (2)	C25—C26—C27—C21	−40.9 (4)
C15—C162—C17—C161	−48.0 (11)	O21—C21—C27—C26	−152.3 (2)
O11—C11—C17—C162	−116.9 (12)	C22—C21—C27—C26	87.4 (3)
C12—C11—C17—C162	6.9 (12)		

### Hydrogen-bond geometry (Å, °)

**Table:**

*D*—H···*A*	*D*—H	H···*A*	*D*···*A*	*D*—H···*A*
O11—H11···O12^i^	0.84	1.92	2.697 (2)	154
O12—H12···O22	0.84	1.90	2.733 (2)	173
O21—H21···O11^ii^	0.84	1.88	2.720 (2)	178
O22—H22···O21^iii^	0.84	1.91	2.699 (2)	156

Symmetry codes: (i) −*x*+1, −*y*+1, −*z*; (ii) *x*, *y*−1, *z*; (iii) −*x*, −*y*, −*z*.
